# Pathological Nuclear Hallmarks in Dentate Granule Cells of Alzheimer’s Patients: A Biphasic Regulation of Neurogenesis

**DOI:** 10.3390/ijms232112873

**Published:** 2022-10-25

**Authors:** Laura Gil, Erika Chi-Ahumada, Sandra A. Niño, Gabriela Capdeville, Areli M. Méndez-Torres, Carmen Guerrero, Ana B. Rebolledo, Isabel M. Olazabal, María E. Jiménez-Capdeville

**Affiliations:** 1 Facultad de Medicina, Universidad Alfonso X el Sabio (UAX), Avenida de la Universidad, 1, 28691 Villanueva de la Cañada, Spain; 2 Departamento de Bioquímica, Facultad de Medicina, Universidad Autónoma de San Luis Potosí, Av. Venustiano Carranza 2405, San Luis Potosí 78210, Mexico; 3 Geroscience Center for Brain Health and Metabolism (GERO), Santiago de Chile 7750000, Chile; 4 Escuela de Medicina, Universidad Panamericana, Ciudad de Mexico 03920, Mexico; 5 Banco de Cerebros (Biobanco), Hospital Universitario Fundación Alcorcón, 28922 Alcorcón, Spain

**Keywords:** hippocampus, Alzheimer’s disease, chromatin markers, nuclear lamin, Tau protein, cell cycle, neurogenesis, nuclear pathology

## Abstract

The dentate gyrus (DG) of the human hippocampus is a complex and dynamic structure harboring mature and immature granular neurons in diverse proliferative states. While most mammals show persistent neurogenesis through adulthood, human neurogenesis is still under debate. We found nuclear alterations in granular cells in autopsied human brains, detected by immunohistochemistry. These alterations differ from those reported in pyramidal neurons of the hippocampal circuit. Aging and early AD chromatin were clearly differentiated by the increased epigenetic markers H3K9me3 (heterochromatin suppressive mark) and H3K4me3 (transcriptional euchromatin mark). At early AD stages, lamin B2 was redistributed to the nucleoplasm, indicating cell-cycle reactivation, probably induced by hippocampal nuclear pathology. At intermediate and late AD stages, higher lamin B2 immunopositivity in the perinucleus suggests fewer immature neurons, less neurogenesis, and fewer adaptation resources to environmental factors. In addition, senile samples showed increased nuclear Tau interacting with aged chromatin, likely favoring DNA repair and maintaining genomic stability. However, at late AD stages, the progressive disappearance of phosphorylated Tau forms in the nucleus, increased chromatin disorganization, and increased nuclear autophagy support a model of biphasic neurogenesis in AD. Therefore, designing therapies to alleviate the neuronal nuclear pathology might be the only pathway to a true rejuvenation of brain circuits.

## 1. Introduction

Maintaining hippocampal synaptic plasticity is crucial for the storage and persistence of memories through brain aging [1,2,3]. Neuronal aging is directly related to DNA damage and disruption of genomic integrity [4,5,6,7,8,9,10,11,12,13] and it is the main cause of neurodegenerative diseases such as Alzheimer’s disease (AD) [14,15,16,17,18,19,20,21]. Considering that hippocampal atrophy is one of the earliest morphological alterations detected in AD by imaging techniques [22,23,24,25], many studies are interested in the progression of these hippocampal changes.

The hippocampus has many different functions, which are carried out through the trisynaptic pathway described by Cajal [26]. This pathway is a complex and dynamic circuit from the entorhinal cortex to the dentate gyrus (DG) and two hippocampal areas, CA1 and CA3. Although these areas are physically close, each presents specific cellular and molecular structure and function [27]. It has recently been described that, under AD conditions, pyramidal CA1 neurons undergo a nuclear transformation that distinguishes them from the neurons of neurologically healthy senile individuals [28,29,30]. These nuclear changes are associated with the aberrant re-entry of post-mitotic pyramidal neurons into the cell cycle [31] and the later development of neurofibrillary tangles (NFTs), toxic cytoplasmic aggregates that characterize intermediate and late AD stages [32]. The pathological hallmarks of aberrant genomic expression in the nucleus of AD neurons are the absence of the chromatin-modifier Tau protein [33,34], the instability of heterochromatin blocs constituting the domains associated with lamin (LADs) and the nucleolus (NADs) [28,35], histone modifications, and a dysfunctional nucleus-cytoplasm restructuration, whereby lamin A expression and altered nucleus-cytoplasm transport through the nuclear pore play an essential role [30,36].

Along with lamin dysfunction [29,30,31] and cytoplasmic Tau pathology [37,38,39], the aberrant chromatin structure of pyramidal AD neurons in humans, rodents, and *Drosophila* AD models is characterized by the dysregulated expression of epigenetic markers H4K20me3 [40], H3K9me3 [41] H3K9me2 [42], and H3K9ac [39]. An altered chromatin pattern has also been reported in cell cultures of fibroblast-derived neurons from AD patients (iNs) with MAPT overexpression [19]. In all cases, nuclear Tau has been closely related to chromatin organization [43,44,45]. In contrast to the regions occupied by pyramidal post-mitotic neurons, the subgranular zone (SGZ) of the DG is one of the few brain areas where neurogenesis persists through adulthood in most mammal species [46]. However, whether this occurs in humans is still under debate [47,48,49,50,51,52,53,54,55]. It has been proposed that new neurons generated in adulthood are involved in adaptive behavioral responses to cognitive and emotional challenges [56,57,58].

The granule cell layer (GCL) displays great cellular heterogeneity determined by its ontogenic origin [59,60,61,62,63]. In rodents, the first granule cells are born in the last embryogenesis stage (embryo granule cells) and the highest rates of postnatal neurogenesis are reached during the first two weeks of life (postnatal granule cells). Though persistent in adult life, the rate of neurogenesis decreases with age [64,65,66]. Neural adult stem cells (NSCs) or quiescent neural progenitors progress to neurons through the proliferation of several intermediate progenitors (amplifiers type 2A and 2B), which lead to neuroblasts or type-3 progenitors after mitotic stages. Neuroblasts differentiate into immature adult-born neurons, which develop axons and ramified dendrites in 4–6 weeks [67,68,69]. Although functionally connected to the tri-synaptic pathway, NSCs are electrophysiologically different from mature granule cells [70]. Their higher excitability and lower threshold for synaptic plasticity suggest that the new and immature adult-born neurons play a unique role in learning and memory within the hippocampal circuit [71].

In rodents, immature adult-born neurons reach morphologic and functional maturity at eight weeks of age, becoming indistinguishable from embryo- and early postnatal-born neurons [72,73,74,75]. In contrast, the maturation process of adult-born neurons of the rhesus monkeys, a species more closely related to humans, lasts up to 24 months [59,76,77]. This extended maturation in primates results in a longer period of favored synaptic plasticity of immature adult-born neurons and an earlier decay of neurogenesis [78,79,80].

Describing nuclear changes throughout senile and AD stages in DG is challenging because this dynamic brain region harbors many immature granular cells in diverse proliferative states [81,82], as opposed to the quiescent pyramidal neurons from the CA1 region [27,83]. In murine models, brain activity related to the exploration of novel environments causes multiple double-strand breaks (DSBs) in DG neurons [84]. However, DSBs are usually repaired within 24 h, especially during sleep [84,85]. Interestingly, DSBs can accumulate in murine AD models, cell cultures, and post-mortem human brain samples obtained from early AD patients [86,87]. These findings have important consequences for DG neurons as the accumulation of DNA damage and failure to repair DNA in proliferating cells cause permanent cell-cycle arrest, senescence, or apoptosis, as predetermined antitumoral mechanisms [88]. In this context, a recent study employing phase-contrast X-ray computed tomography of post-mortem AD human hippocampi showed important nuclear alterations in granular DG cells [89].

This study analyzed the progression of nuclear pathology in DG neurons at early, intermediate, and late AD stages compared with healthy aged and senile conditions. Similar to previous studies in pyramidal CA1 neurons [28], we examined the expression of the chromatin markers phospho-Tau AT100 y AT8, the components of nuclear lamin (NL), and epigenetic histone modifications related to chromatin condensation. In light of recent reports of lamin degradation by autophagy as a response to genotoxic insult [90], we also explored autophagy as a component of nuclear AD pathogeny [91,92,93,94].

## 2. Results

### 2.1. Nuclear Changes in the Entorhinal Cortex at Early AD Stages

**Figure 1:**
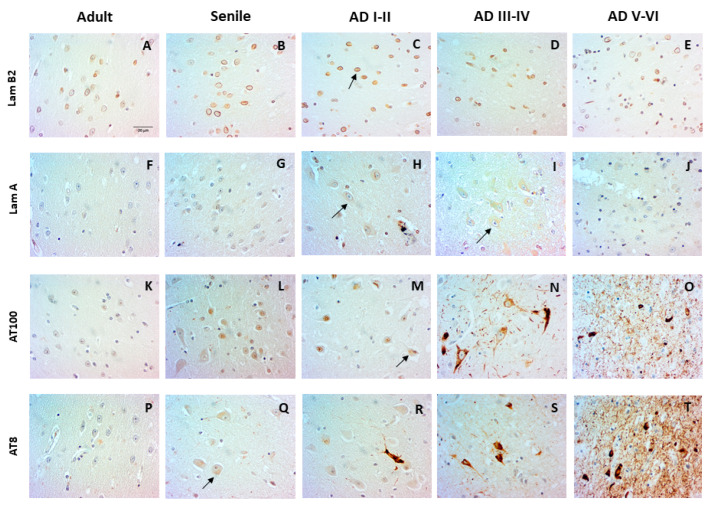
Changes in pyramidal neurons of layer II of the entorhinal cortex. Perinuclear and nucleoplasmic (arrow) Lamin B2 (**A**–**E**). Lamin A absence in adult and senile stages (**F**,**G**) and presence in AD I–IV stages (**H**,**I**) and absence in AD V–VI stages (**J**). Nuclear Tau, AT100 (**K**–**M**), AT8 (**Q**) cytoplasmic and extracellular AT100 and AT8 (**N**,**O**,**R**–**T**). Absence of nuclear Tau AT8 (**P**). Scale bar: 20 µm.

Layer II pyramidal neurons of the entorhinal cortex are the main input to DG granular neurons. We observed that these pyramidal neurons displayed AD-associated changes that followed a similar time course to those reported in pyramidal hippocampal cells. Figure 1 shows the redistribution of Lamin B2 (Table 1) to the nucleoplasm, which started in the senile stage and increased in AD I–II stages (A–C). Increased lamin B2 expression was accompanied by the expression of Lamin A (Table 1) from early to late AD stages (H–J). The shift of phospho-Tau from the nucleus to the cytoplasm preceded NFT formation. The presence of AT100 (Table 1) in the nucleus increased in senile subjects (L) and constituted cytoplasmic NFTs in AD III–VI (N–O). Moreover, AT8 (Table 1) first appeared in the nucleus of senile neurons (Q) and formed NFTs from I to VI AD stages (R–T).

### 2.2. The Distribution of Lamin B2 Uncovers Two Populations of DG Granular Neurons

**Figure 2:**
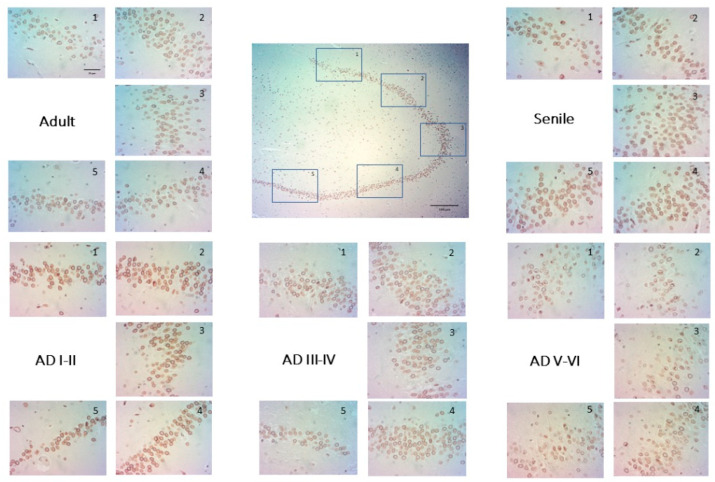
Lamin B2 distribution in the nuclei of DG neurons. Center: Panoramic view of human DG indicating fields (**1**–**5**) of microphotographs and digital quantification of immunopositivity. Scale bar: 100 µm. Five representative microphotographs of adult, senile, and AD stages I–VI. Scale bar: 20 µm. Intermediate and late AD stages (III–VI) show the highest amount of Lamin B2 positivity limited to perinuclear borders.

Lamin B2 plays an important role in chromatin organization and nuclear architecture. It forms a protein meshwork on the nucleoplasmic side of the nuclear membrane. Lamin B2 staining in granular DG neurons was found in thin layers surrounding the nucleus in some cells to the whole nucleoplasm in others. The proportion of these two types of nuclear Lamin B2 neurons varied drastically across age and AD disease progression. In healthy adults, perinuclear Lamin B2 predominated (Figure 2A), but senile and AD I–II stages were characterized by immunopositive nucleoplasms (Figure 2, senile, AD I–II). In contrast, intermediate and late AD stages showed mostly perinuclear Lamin B2 staining around clear nucleoplasms (Figure 2, AD III–IV, AD V–VI). As expected, no Lamin A expression was detected in any condition, given its normal regulation through miR-9 in healthy neurons [11,12].

### 2.3. Phosphorylated Tau (AT100 and AT8) Is Absent in the DG Cell Nucleus at Intermediate and Late AD Stages

**Figure 3:**
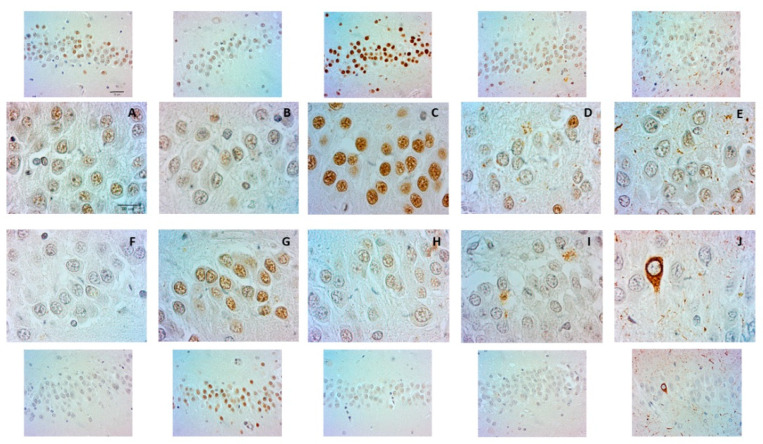
AT100 and AT8 in DG granule cells. Detail of AT100 transition from nuclei (**A**–**C**) to cytoplasm and extracellular sites in AD III–VI (**D**,**E**). Scale bar: 10 μm. Lower magnification microphotographs (upper panels) show the panoramic view of AT100 immunopositivity. Scale bar: 20 μm. AT8 is scarce in adult DG (**F**) and shows a strong nuclear increase in senile DG (**G**). Nuclear positivity gradually disappears from AD I–II to AD V–VI (**H**–**J**), and even an NFT can be observed (**J**). Lower magnification microphotographs (lower panels) show panoramic views of AT8 immunopositivity.

Nuclear immunopositivity to AT100 was present in adult and senile DG cells and increased notably at early AD stages (Figure 3A–C). At AD III–IV, AT100 staining was no longer homogenous; instead, it faded and concentrated in nuclear puncta. Moreover, extranuclear AT100 was observed in the neuropile and some cells still showed nuclear positivity (Figure 3D). Scarce nuclear positivity and increased extracellular AT100 staining were accentuated at late AD stages (Figure 3E).

Strong AT8 nuclear positivity appeared only in senile DG neurons and started fading in AD I–II (Figure 3F,G). From AD III to AD VI, AT8 nuclear positivity was no longer observed. Furthermore, neuropile aggregates and even a few NFTs were found at late AD stages (Figure 3H–J). The quantification of nuclear AT100 and AT8 immunopositivity showed the lowest levels in late AD stages (Figure 3C,D).

### 2.4. AD Granular Neurons Show Drastic Changes in Epigenetic Chromatin Markers

**Figure 4:**
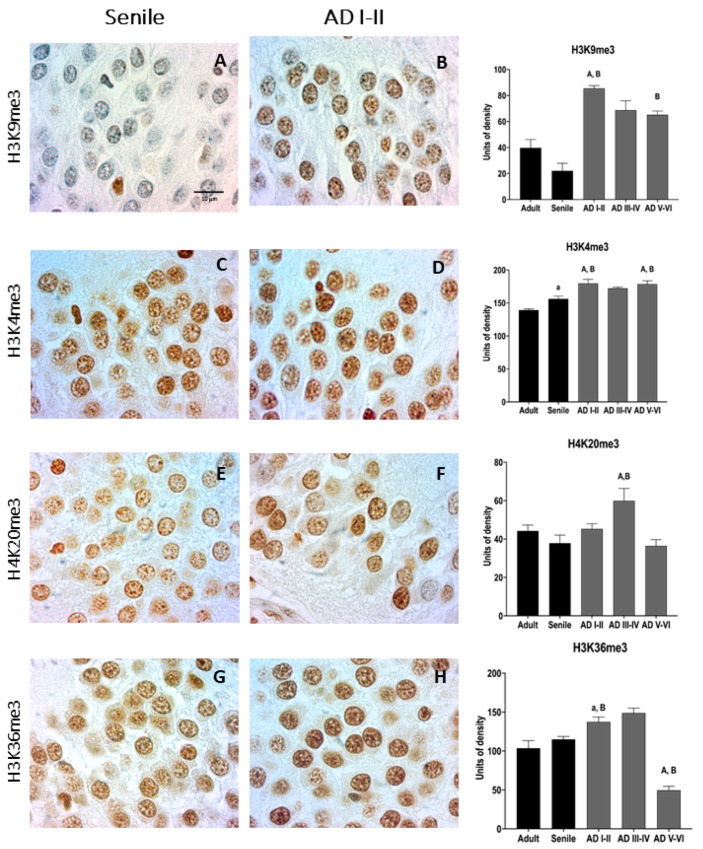
Representative microphotographs of chromatin markers at senile and AD I–II stages. The greatest difference is observed with the H3K9me3 marker (**A**,**B**). A slight increase is detected with the rest of the markers: H3K4me3 (**C**,**D**); H4K20me3 (**E**,**F**) and H3K36me3 (**G**,**H**). Scale bar: 10 μm. Quantification bars (mean + SD) show statistically significant differences versus adult (A, *p* < 0.001, a, *p* < 0.05) and senile subjects (B, *p* < 0.001).

As reported in the literature, the suppressive mark H3K9me3 (Table 1) increased sharply at early AD stages and remained elevated until late stages (Figure 4A,B). A similar pattern was observed in H4K20me3 (Figure 4E,F and Table 1). Chromatin markers H3K4me3 (Figure 4C,D) and H3K36me3 (Figure 4G,H) (Table 1), which are associated with increased genic expression, also changed from senile to AD I–II, indicating complete chromatin remodeling of DG neurons.

### 2.5. Intermediate and Late AD Stages Are Characterized by Increased Nuclear and Perinuclear Autophagy Marker (LC3)

**Figure 5:**
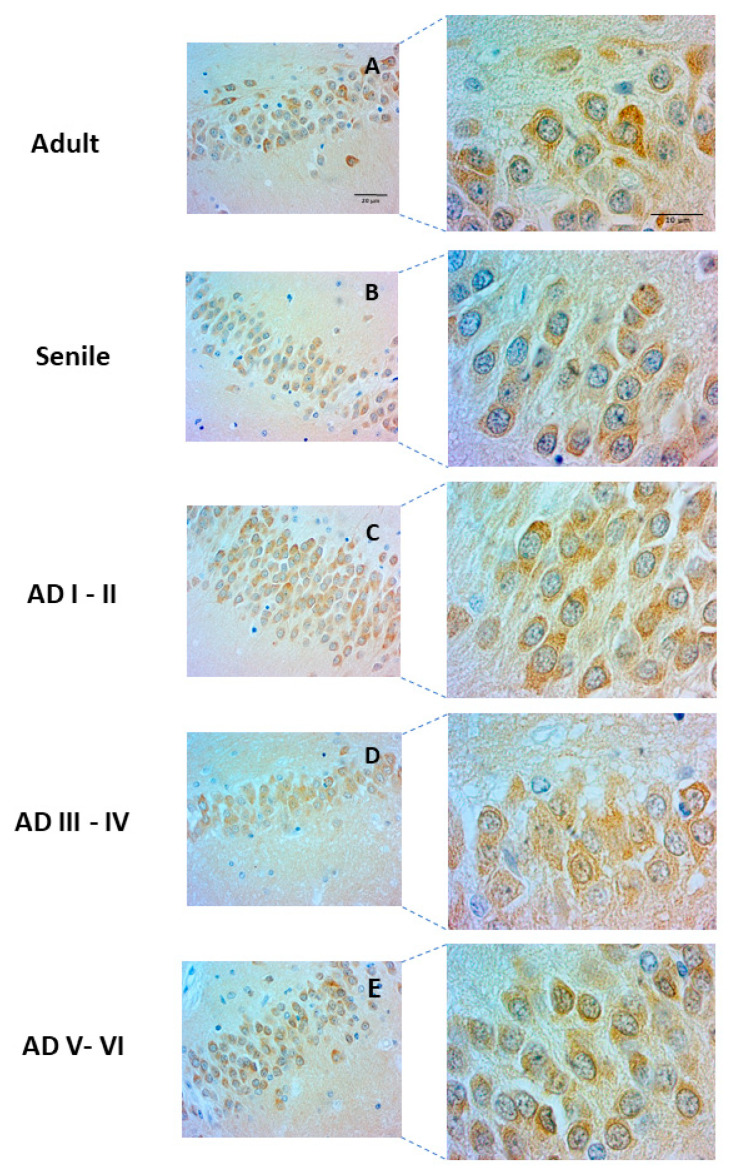
Increased nucleophagy in intermediate and late AD stages. Transition from predominantly cytoplasmic (**A**–**C**) to nuclear and perinuclear LC3 immunopositivity (**D**,**E**). Scale bar: 20 µm. Right panels show higher magnification of the nuclear LC3 autophagy marker, noticeable in intermediate and late AD stages. Scale bar: 10 µm.

**Figure 6:**
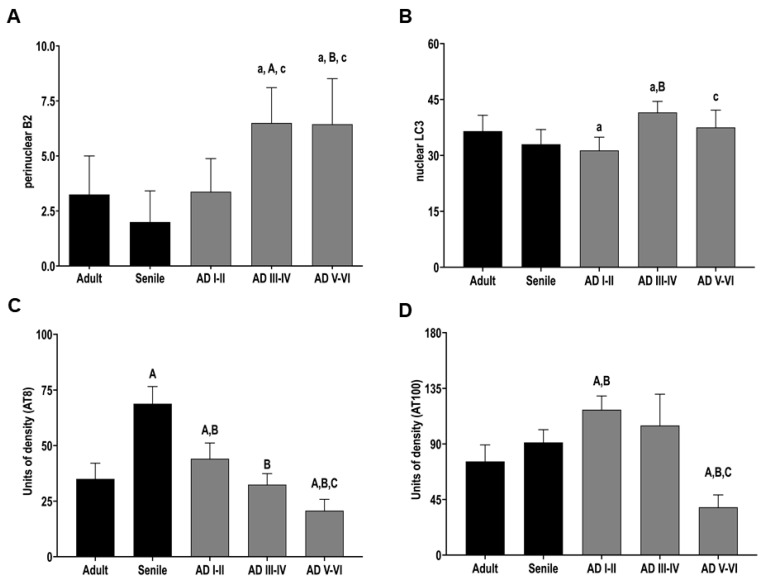
Quantification of AD hallmarks in DG cells. Perinuclear Lamin B2 (**A**) and nuclear LC3 immunopositivity (**B**) show significant increases in intermediate and late AD stages. These changes concur with decreased AT8 nuclear levels (**C**) and AT100 (**D**). Statistically significant differences versus adult (A, *p* < 0.001, a, *p* < 0.05), senile subjects (B, *p* < 0.001), and AD I-II (C, *p* < 0.001, c, *p* < 0.05). Each bar represents the mean + SD.

We found a striking spatial rearrangement of LC3 (Table 1) from the healthy to the AD condition and across AD stages. In healthy and early AD stages, LC3 was mainly localized in the cytoplasm of DG cells (Figure 5A–C); however, this autophagy marker increased in the nucleus and perinucleus from AD III to AD VI (Figure 5D,E), suggesting a nucleophagy phenomenon. The increased nuclear LC3 immunopositivity is concomitant with the disappearance of nucleoplasmic Lamin B2 (Figure 5E,F). In agreement with this, nuclear L3 increases in a similar manner to perinuclear Lamin B2 (Figure 6A,B).

## 3. Discussion

This study describes the nuclear pathology of DG neurons throughout AD stages as compared with aging-related changes. We found nuclear alterations in granular cells different from those reported in pyramidal neurons from CA1 and CA3 hippocampal regions. However, pyramidal neurons from layer II of the entorhinal cortex, which are the main input to the DG, also present the nuclear pathology previously described in hippocampal subfields of AD brains [28]. We describe important changes in nuclear lamin B2 distribution across different AD stages compared with healthy DG cells. These changes occur alongside the disappearance of phosphorylated Tau forms from the nuclei and, interestingly, with increased nuclear autophagy markers in intermediate and late AD stages. As a hallmark of chromatin transformation from a healthy to a pathologic state, the remarkable increase in the epigenetic markers H3K9me3 and H3K4me3 clearly delineates the limit between aging and AD in granular cells. In the context of the abundant data on adult human neurogenesis (AHN), these results obtained in the dynamic niche of DG neurons suggest a biphasic model for the regulation of neuronal survival through AD.

### 3.1. Similarities between Changes in CA1 and Entorhinal Cortex Pyramidal Cells in AD

The tri-synaptic circuit contains three regions with post-mitotic pyramidal neurons containing big nuclei and extensively ramified dendritic trees [22]. However, neuropathological data suggest that they have a different vulnerability to aging and AD [23,95]. Pyramidal neurons from these three regions accumulate nuclear Tau (phosphoepitope AT100) associated with aging, which constitutes a hallmark of aged chromatin [96]. Nuclear Tau is a global genome stabilizer, especially of heterochromatin blocs NADs and LADs [33,97,98]. Oxidative DNA damage associated with neuronal aging triggers the exit from the quiescent state and the aberrant re-entry into the cell cycle [99,100]. The transformation of a healthy senile brain to AD pathology inaugurates the coexistence of two types of pyramidal neurons. One population expresses nucleoplasmic lamin B2, a sign of active transcription and abortive cell cycle that leads to neuronal death [31,101,102,103,104], and the population of AD neurons, characterized by an anomalous lamin A expression [28,29,30,105], which stops the cell cycle, thus protecting the nucleus and generating a new cytoskeleton [106,107,108]. Early AD stages are also distinguished by the gradual disappearance of nuclear AT100 [34], which is strongly associated with chromatin disorganization [29].

### 3.2. The Dynamic Neuronal Nature of DG through Development and Aging

The DG is considered the “gateway” to the hippocampus that introduces different cellular and functional characteristics into the tri-synaptic circuit. The GCL includes (i) small post-mitotic mature neurons with short dendritic trees and different ages [109]; (ii) immature newborn granule neurons in different proliferative stages [110]; and (iii) quiescent radial glia cells, which generate the latter group and are located in the subgranular zone (SGZ) niche [111]. Notably, the number of immature granule newborn cells that the GCL incorporates is not constant; rather, it changes depending on internal and external factors [112,113,114,115,116]. In humans, the persistence of AHN [54,55,56] is supported by BrdU (5-bromo-2′-deoxyuridine) incorporation and ^14^C quantification in proliferating neurons [48,49]. However, the human GCL shows more immature neurons than other non-primate mammals [52,76,117].

Aging is one of the endogenous factors that account for the progressively slower incorporation of newborn mature neurons into the GCL [67,118]. These cells gradually replace neurons generated throughout development [49]. Compared with other hippocampal regions, the DG shows less aging-associated neuronal loss [54,55,68] and a lower decline rate [119,120,121,122,123,124]. DG neurogenesis occurs in five phases characterized by the expression of specific cell markers [27,84,85,86,87,88]: proliferative (stage 1), differentiation (stage 2), migration (stage 3), targeting (stage 4), and synaptic integration (stage 5) [50]. From the proliferative point of view [110], immature newborn granular neurons in stages 1, 2, and 3 are in a replicative cycle. In contrast, stage 4 granular neurons have differentiated into a post-mitotic state, associated with the temporal expression of calcium-binding protein calretinin [125,126,127]. Finally, stage 5 synaptically mature neurons express calbindin [50].

Our results indicate that nuclear phenotypes of senile GCL neurons are slightly different from those of adult brains regarding nuclear lamin structure and chromatin organization. In rat GCL, lamin B2 positivity increases throughout neurogenesis, reaching its highest level in mature neurons [128]. In human GCL, however, B2 positivity can be found in the perinucleus, similar to senile pyramidal neurons [28], as well as in the nucleus. B-type lamins redistribute from the nuclear envelope to replication factories during S-phase [129] and modulate nucleolar function [130]; therefore, the presence of B2 lamin in the nucleus suggests that the cells are in a replicative cycle, probably as newborn granular neurons in the first maturation stages (1, 2, and 3). In contrast, the cells with strong perinuclear positivity [106] would be either (i) newborn post-mitotic granular neurons undergoing different axonal and dendritic development (stages 4 and 5) [71,131] or mature neurons already structurally and functionally integrated into the hippocampus. The significantly higher proportion of perinuclear positivity in intermediate and late AD stages indicates fewer immature neurons, lower neurogenesis, and fewer adaptations to environmental factors.

### 3.3. Heterochromatin Markers and Nuclear Tau: Hallmarks of Early AD in Granular Cells

DG cells with aged chromatin cannot properly repair DSBs [132]. DSBs are involved in the synaptic activity required to explore novel environments [84,85]; thus, the accumulation of DNA mutations affects genome integrity [20,133]. Our results show that phosphorylated Tau is increased in the nuclei of senile GCL cells, similar to previous reports of pyramidal neurons from CA1, CA3 [28,96], and EC (Figure 1). Tau plays an important role in the protection against DNA damage [134,135]. Moreover, DSBs’ induction immediately increases Tau in the nuclear fraction of primary mouse cortical neurons [136]. The phosphorylation of the AT8 site is specific for senile chromatin [28] and has been directly related to DSBs’ induction in cortical neurons [136]. In granular neurons, the double AT100 and AT8 presence seems to determine the nuclear Tau function interacting with aged chromatin, favoring DSBs’ repair [136,137] and maintaining genomic stability [43,138]. This stabilization would compensate for the decrease in the constitutive heterochromatin marker H3K9me3 [139,140,141], which is fundamental for the structural organization of NADs and LADs [142] and genomic integrity [143].

In this context, as senile GCL preserves the number of functional neurons [144,145,146,147], the regression of dendritic trees and synaptic dysfunction would be more closely related to the observed age-related cognitive decline [55,147,148,149]. What molecular events in the GCL dynamic niche could explain the dysfunctional communication progressing through AD?

Using three-dimensional histology based on phase-contrast computed tomography, Eckermann and collaborators recently reported a decreased nuclear volume of GCL cells in AD as compared with healthy brains, which was related to a higher ratio of heterochromatin to euchromatin [89]. In agreement with these observations, we found histological evidence of the upregulation of the constitutive heterochromatin epigenetic marker H3K9me3 [139,150]. Furthermore, H3K4me3 is a hallmark of the GCL neuronal transformation from senile to AD. Similar increments of H3K9me3 have been reported in cortex motor neurons of AD and Huntington’s disease patients [151,152]. H3K9 trimethylation maintains the structural heterochromatin domains and contributes to silencing repetitive sequences of peri- and centromeric regions [150] and stabilizing most of the long interspersed nuclear elements and endogenous retroviruses [153]. Conversely, the absence of H3K9me3 dissolves heterochromatin [139], leading to genomic instability and chromatin disorganization [143]. Based on an integrated analysis of genome-wide ChiP- and mRNA-sequencing, abnormal heterochromatin remodeling by increased neuronal H3K9me3 expression results in down-regulation of (i) synaptic function, (ii) neuronal differentiation, and (iii) cell motility-related genes [114]. In the human GCL, the transformation of senile granular neurons to the AD phenotype implies high H3K9me3 expression along disease progression, reaching its highest level at early AD stages (AD I–II). Nevertheless, the exact role of increased H3K9me3 is still controversial as it may be affecting populations in different maturity states. In vivo reprogramming of murine aging models to elevate H3K9me3 in the DG showed that it increases the survival of newborn DG neurons and improves memory functions [154].

Nuclear alterations, including NL reinforcing and heterochromatin stabilization in the absence of cytoplasmic pathology, characterize early AD in CA1, CA3, EC pyramidal neurons, and DG cells. These hippocampal functional changes are associated with learning and memory alterations and AHN as an adaptation to hippocampal functioning [127]. This AHN is similarly stimulated by pathologies like hippocampal or cortical damage associated with ischemic insults or epileptic seizures [155,156,157,158,159,160]. In this context, several groups reported increased neurogenesis and proliferation markers in murine models and human AD, such as doublecortin (DCX-labeled immature neurons) [161,162,163], calretinin (CR), and Ki67 [164]. Given that the levels of DCX and CR increase in early AD compared with late AD stages, an AHN increment might be a characteristic response to the transformation from senile to AD neurons [55]. Our results support AHN at early AD stages because we observed the upregulation of the epigenetic silencer H3K9me3 along with an abrupt increase in H3K4me3 expression. As H3K4me3 is considered a sign of actively transcribed promoters of cellular function/identity, we believe that these cells are under active transcription [165].

Both post-transductional trimethylations of H3 are the highest at the early AD stages and slightly decline at the late stages (Figure 4). In this context, it is remarkable that, during the intermediate and late AD stages, the DG shows more neurons with perinuclear lamin B2 than in adult, senile, and even AD I–II stages. These nuclei with reinforced NL and less active transcription might correspond to the pool of newborn granular post-mitotic neurons with a suboptimal development of their dendritic tree and affected synaptic connections [114,129]. Further experiments will confirm the identity of this cell population with increased perinuclear lamin B2. Nevertheless, the increase in post-mitotic cells in AD III–IV would result from a higher rate of cells entering the cell cycle, coinciding with the upregulation of AT100 at early AD stages. As mentioned before, nuclear Tau protects the structural integrity of condensed chromatin [100,101] and has been widely reported in the nucleolus of human hippocampal neurons and cultured cells [28,34,96,166,167].

Hence, we propose that the synchronic nuclear pathology throughout the tri-synaptic circuit profoundly affects the regulation of the neurogenic niche, leading to a robust increase in immature cells, DCX+, and CR+, which could represent compensation for neuronal damage and loss [28].

### 3.4. Increased Nuclear Autophagy in Late AD Stages Supports Biphasic Neurogenesis Changes in AD

The higher numbers of DCX+ and CR+ granular cells detected at the III–IV AD stages [55,161,162,164] have also been reported in other neuropathologies such as epilepsy, schizophrenia, and Creutzfeld–Jacobs disease [164,168,169,170,171]. This situation has led pathologists to coin a new term to define this peculiar state: “immature dentate gyrus” (iDG) [168]. However, this proliferation boost does not generate a net increase in granular post-mitotic neurons in maturation stages at intermediate and late AD stages [55,161,162]; rather, the number of granular DG cells remains relatively stable [164]. In epilepsy, for example, this “partial rejuvenation” process is followed by aberrant maturation and integration into the tri-synaptic circuit, which contributes to the further development of spontaneous and frequent seizures [171,172]. Contrarily, our results show, for the first time, that intermediate and terminal AD stages are characterized by nuclear degeneration through autophagy-based lamin B2 degradation, known as nucleophagy [90,173].

Autophagy is essential to induce senescence after oncogenic activation derived from DNA damage [174]; this mechanism involves lamin B1 nucleophagy [175]. Autophagy is also considered a common trait of neurodegenerative diseases like AD [176,177]. Accordingly, increased nucleophagy has been reported in patients with dentatorubral-pallidoluysian atrophy (DRPLA), one of the diseases associated with poliglutamine (polyQ) repeats, in which affected individuals develop symptoms of ataxia, epilepsy, and dementia [178]. Functional autophagy seems to prevent neuronal senescence [179]. In our study, increased nucleophagy in the DG overlaps with nuclear depletion of AT 100 at intermediate AD stages. In fact, the loss of nuclear Tau function and the consequent heterochromatin disruption have been suggested as the actual triggering factors for AD [138]. Chromatin disorganization is initiated by nuclear Tau downregulation and lamin B2 disruption, which involves the disappearance of the nucleoplasmic NL structure [180]. Under these conditions, the heterochromatin markers H3K9me3 and H4K20me3 still hold back the phenomenon, and transcription is still activated, as indicated by increased H3K4me3 and H3K36me3 markers. However, in late AD stages (V–VI), chromatin becomes significantly disorganized, as indicated by the decreased epigenetic markers, putting GCL neurons to the same end as pyramidal cells of the tri-synaptic circuit [29] and neurons from the transgenic *Drosophila* and mouse AD models [37,38].

## 4. Materials and Methods

### 4.1. Human Brain Samples and Immunohistochemistry

Human brain tissue samples were collected at the Tissue Biobank of the Hospital Universitario Fundación Alcorcón (HUFA), C/ Budapest 1, 28922 Alcorcón, Madrid, España. The protocol was reviewed and approved by the institutional Clinical Research Ethics Committee of HUFA (47/2018, in October 2018). Samples were obtained from four healthy subjects of different ages (37, 42, 65, and 76 years) with no evidence of cognitive impairment or dementia and six AD cases diagnosed post-mortem according to Braak’s classification. To classify the specimens, we used the characteristics of I–VI AD stages updated for paraffin sections and immunocytochemistry by Braak and collaborators in 2006 [180]. Three samples presented early stages I–II (ages 68 and 77), two intermediate stages III–IV (72 and 82), and two late stages (79 and 89). Paraffin sections obtained at the DG level of the hippocampus were processed for immunohistochemistry with the following antibodies.

The immunohistochemical assessment was performed on 4 µm thick dewaxed sections. After boiling the sections in a pressure cooker with DIVA decloaking solution (Biocare Medical, LLC, Concord, CA, USA) for epitope recovery, endogenous peroxidases were blocked with Dako Peroxidase Blocking Reagent (DAKO, Glostrup, Denmark). Next, diluted primary antibodies were incubated overnight at 4 °C. After incubation with the primary antibody and buffered phosphate rinses (PBS), sections were exposed to the streptavidin-biotin marked secondary antibody. The peroxidase reaction was visualized with 3′3-diaminobenzidine. The sections were finally counterstained with hematoxylin (HE), dehydrated, and cover-slipped for microscopic observation. The sections were observed on an Olympus microscope equipped with a digital camera (Amscope, Irvine, CA, USA).

### 4.2. Image Acquisition and Analysis

DG section and image serial photomicrographs at 20X, 40X, and 100X were obtained all along the DG structure. The total number of microphotographs (16 bit) inspected ranged from 40 to 60 per subject. All image analyses were performed on raw data.

Image blinding: For each antibody, all images were taken on the same day. Collected images were stitched together using Stitching plug-in of Fiji/Image J2 version 2.9.0/1.53t; Java 1.8.0_202 (Wayne Rasband, Bethesda, MD, USA). Image files were each assigned a random alphanumeric code to ensure that the subsequent steps in image segmentation were blind to diagnosis (control vs. AD) and donor identity.

Background subtraction: To minimize background differences across sections, the contrast and brightness were adjusted equally for all images within a series. The rolling ball Fiji algorithm was used with a radius of 200 pixels. Non-specific background staining was subtracted from the measured values.

Region of interest segmentation and processing: A specific built-in algorithm called color deconvolution was used [181]. This algorithm separated the staining of HE and DAB into three different panels, namely, HE (panel 1), DAB-only image (panel 2), and background (panel 3). Panel 2 was converted to grayscale for threshold selection and was auto-thresholded using the Otsu Fiji method and converted into a mask [28]. The area was selected (region of interest, ROI) by adjusting the brush size to the neuron soma, with a minimum cell size of 100 pixels. Masks were then eroded to separate the nucleus. The mean optical density of the immunopositive cells was calculated and pixel intensities were maintained within a linear range to ensure accurate quantification.

To quantify the LC3 nuclear area, the threshold was established in the images and particles were evaluated within defined circular ROIs of 10 µm in diameter per picture/field/subject and compared to the global average. Only particles larger than 0.5–1.5 pixels were selected by the algorithm CellProfiler 2.2.0 (https://cellprofileranalyst.org/, accessed on 9 June 2021). Aggregate analysis was verified by manual inspection of each neuron at 100X magnification. Finally, images containing errors in focus, tracing, or other artifacts, such as lint fragments or dye precipitates, were omitted from the analysis.

### 4.3. Data Analysis

All data were analyzed for normality and variance homogeneity and several tests were used for statistical comparison. Statistical calculations were carried out in Prism 9.4.1 (GraphPad Software, San Diego, CA USA, www.graphpad.com, accessed on 9 June 2021) Adult, senile, and AD groups were compared with either a one-way ANOVA followed by Dunnett’s test or a Kruskal–Wallis test followed by the Mann−Whitney U test in order to identify significant differences (* *p* < 0.05) versus the control group.

## 5. Conclusions

Similar to prion disease [164], our results suggest a biphasic model of neurogenesis regulation in AD [182]. Nuclear alterations in all regions of the tri-synaptic circuit induce a strong neurogenesis reactivation in DG. These findings, together with the evidence of substantial transcriptional heterogeneity within the hippocampus [183], could be related to the lower vulnerability of DG to oxidative stress, caloric restriction, and different insults [1,184,185,186,187,188,189].

The elevated number of granular cells, immature and without functional chromatin, do not integrate into the GCL, but suffer nucleophagy at intermediate and late AD stages. Although somehow in disagreement with the data presented by Tobin and collaborators [55], the laminar and chromatin pathology affecting all granular neurons (namely, old mature, newborn immature, and those generated during “AD neurogenesis”) has negative consequences on DG gene transcription, synaptic plasticity, and integrated cognitive functions [13]. In summary, our results suggest that generalized nuclear pathology plays a central role in the neurogenic state of the DG through aging and early AD stages, which later progresses into increasingly severe genomic dysfunction. This dysfunction generates progressive cognitive deficits determined by the neuron’s different vulnerability and connectivity, whereby cytoplasmic pathology plays a delayed role.

## 6. Future Perspectives

Dysfunction of all areas of the tri-synaptic hippocampal circuit converges in impaired AHN, leading to severe cognitive deficits in AD brains. The idea that an increased number of adult-generated neurons could ameliorate cognitive impairment [190] becomes questionable in light of the aberrant neurogenesis observed at early AD stages [191], perhaps as a result of neuron subtype 2 (SOX-) proliferation [192]. Regardless of the quiescent or replicative nature of granular GCL neurons, EC, CA1, and CA3 pyramidal neurons, their nuclear pathology appears simultaneously in the damaged hippocampus. All neurons generate different versions of a closed chromatin structure and a rigid nucleoskeleton. Therefore, the crucial point is that aging induces chromatin transformations that drive cells to an aberrant cell-cycle re-entry in order to repair their genome. This process finally leads to cancer or degeneration. Designing therapies to alleviate the neuronal nuclear pathology might be the only pathway to a true rejuvenation of brain circuits.

## Figures and Tables

**Table 1:** List of employed antibodies.

Antibody (Clone)/Supplier/Catalog Number/Manufacture
Anti-Lamin A antibody/Abcam/ab26300/Cambridge, UK Species: Rabbit Polyclonal Dilution: 1:500 Anti-Lamin B2 antibody [EPR9701(B)]/Abcam/ab151735/Cambridge, UK Species: Rabbit Monoclonal Dilution: 1:200 Anti-Human Phospho-PHF-Tau pSer202/Thr205 (AT8)/Thermo Fisher Scientific/MN1020/Rockford, IL, USA Species: Mouse monoclonal Dilution: 1:20 Phospho-Tau (Thr212,Ser214) (AT100)/Invitrogen, Thermo Fisher Scientific/MN1060/Rockford, IL, USA Species: Mouse Monoclonal Dilution: 1:100 Anti-Histone H3 (tri methyl K9) antibody [6F12-H4]/Abcam/ab184677/Cambridge, UK Species: Mouse Monoclonal Dilution: 1:100 Anti-Histone H4 (tri methyl K20) antibody/Abcam/ab195479/Cambridge, UK Species: Rabbit Polyclonal Dilution: 1:100 Anti-Histone H3 (tri methyl K4) antibody/Abcam/ab213224/Cambridge, UK Species: Rabbit Monoclonal Dilution: 1:100 Anti-Histone H3 (tri methyl K36) antibody/Abcam/ab9050/Cambridge, UK Species: Rabbit Polyclonal Dilution: 1:100 Anti-LC3B antibody [EPR18709]—Autophagosome Marker/Abcam/ab192890/Cambridge, UK Species: Rabbit Monoclonal Dilution: 1:100

## Abbreviations

AD	Alzheimer’s disease
AHN	adult human neurogenesis
AT8	Tau protein phosphorylated at Ser202/Thr205
AT100	Tau protein phosphorylated at Thr212/Ser214
BrDU	5-bromo-2′-deoxyuridine, proliferation marker
CA1	Ammon’s horn region 1 (hippocampal region with pyramidal cells)
CA3	Ammon’s horn region 3 (hippocampal region with pyramidal cells)
CR	calretinin protein, neurogenesis marker
ChIP	chromatin immunoprecipitation
DCX	doublecortin protein, neurogenesis marker
DG	dentate gyrus
DRPLA	dentatorubral-pallidoluysian atrophy
DSBs	double strand breaks
EC	entorhinal cortex
GCL	granule cell layer
H3K9me3	trymethylated Histone 3 protein, heterochromatin marker
H4K20me3	trymethylated Histone 4 protein, heterochromatin marker
H3K4me3	trymethylated Histone 3 protein euchromatin marker
H3K36me3	trymethylated Histone 3 protein euchromatin marker
iDG	immature dentate gyrus
iNs	induced neurons
Ki67	proliferation marker
LADs	lamin associated domains
LC3	autophagy marker (protein MAP1LC3B)
MAPT	Tau protein
NADs	nucleolus-associated domains
NL	nuclear lamin
NFT	neurofibrillary tangles
NSC	neural stem cells
PolyQ	polyglutamine repeats-associated disease
SGZ	subgranular zone
